# Hemichorea-Hemiballism Secondary to Non-Ketotic Hyperglycemia

**DOI:** 10.14740/jocmr2259w

**Published:** 2015-07-24

**Authors:** Jordan E. Pinsker, Keivan Shalileh, Veronica J. Rooks, Richard W. Pinsker

**Affiliations:** a William Sansum Diabetes Center, Santa Barbara, CA, USA; b Jamaica Hospital Medical Center, New York, NY, USA; c Tripler Army Medical Center, Honolulu, HI, USA

**Keywords:** Hyperglycemia, Dyskinesias, Chorea

## Abstract

Non-ketotic hyperglycemia is an unusual and rare cause of hemichorea-hemiballismus. Correction of the hyperglycemia usually results in total resolution of the signs and symptoms. We present the case and medical imaging findings of a 66-year-old female who presented with steadily worsening choreiform and ballistic movements of the right upper and lower extremities over a 2-week period. Her serum glucose was greater than 600 mg/dL, and no ketones were present. CT scan and MR demonstrated left basal ganglia abnormalities suggesting hyperglycemia-related hemichorea-hemiballismus syndrome. Restoration of euglycemia led to eventual resolution of all symptoms. Knowledge of this disorder is paramount so as to rule out other causes of intracranial pathology.

## Introduction

Hyperglycemia is a rare cause of movement disorders. We present the case and medical imaging findings of a 66-year-old female who presented with worsening choreiform and ballistic movements secondary to non-ketotic hyperglycemia that subsequently improved with insulin treatment.

## Case Report

A 66-year-old female presented to the emergency department with steadily worsening purposeless involuntary movements of the upper and lower right extremities over the prior 2 weeks. Two days prior, the patient had visited the emergency department and was found to have a serum glucose greater than 600 mg/dL and no ketonemia. Prior to this she had no known history of diabetes. She briefly received insulin and intravenous fluids but was sent home on metformin and sitagliptin when she declined to use insulin at home. She then returned with worsening involuntary movements.

**Figure 1:**
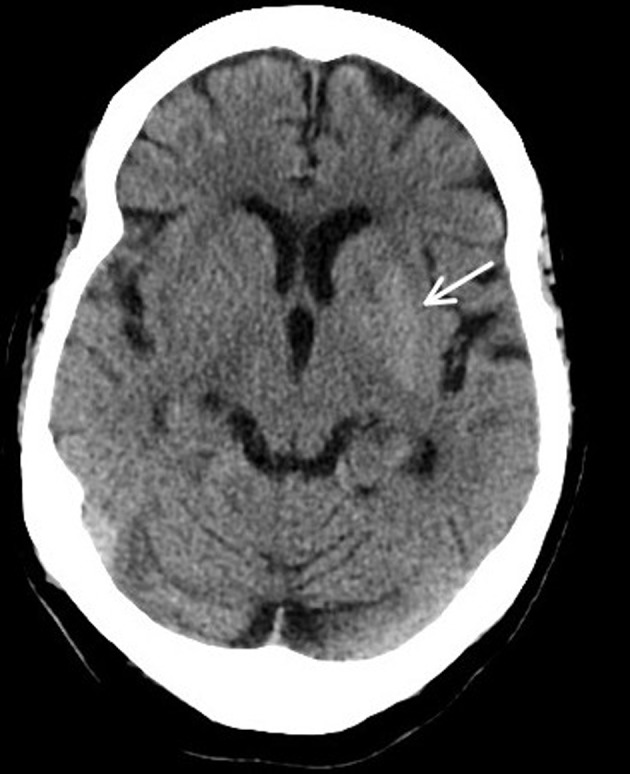
Axial CT demonstrated increased density within the caudate nuclei and left lentiform nucleus (arrow).

**Figure 2:**
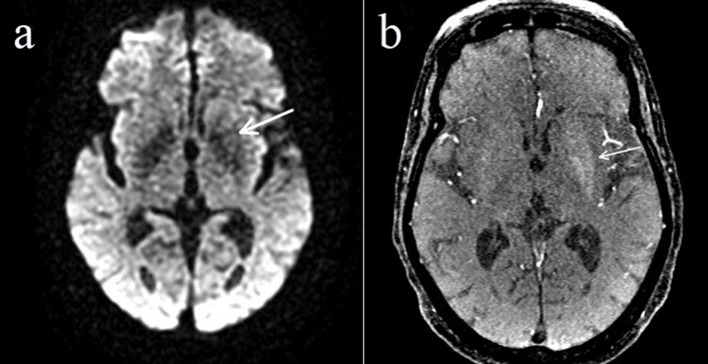
There was minimal increased signed intensity on the diffusion-weighted imaging sequence (arrow) (a), as well as T1 shine through on the time of flight sequence (arrow) (b).

Physical examination was remarkable for choreiform and ballistic movements of the right upper and lower extremities. These movements did not lessen with sleep. Laboratory evaluation again showed severe hyperglycemia without evidence of ketonemia. CT scan and MR with diffusion-weighted imaging showed abnormalities in the left basal ganglia suggesting possible hyperglycemia-related hemichorea-hemiballismus syndrome with no evidence of stroke (Fig. 1, 2). Aggressive insulin treatment led to eventual resolution of her abnormal movements, although they did not fully resolve until almost 1 month later.

## Discussion

Although uncommon, hemichorea-hemiballismus syndrome due to non-ketotic hyperglycemia can be the first presenting sign of diabetes mellitus or can occur after many years of poor glycemic control [1]. Women are affected more than men, with a mean age of onset of 72 [2]. This syndrome has also recently been reported in adolescents with new onset diabetes [3]. CT scan typically shows an area of hyperdensity in the basal ganglia, with no associated mass effect, edema, or other signs of hemorrhage. MRI can show high T1-weighted signal in the same area [4]. Cerebral ischemia with resulting dysfunction of GABAergic projection neurons has been proposed as a possible mechanism for these findings [5].

Resolution of symptoms usually occurs rapidly with restoration of euglycemia, although in some instances, similar to our patient, the symptoms may persist for some time [6]. Since timely restoration of euglycemia can result in rapid and complete resolution of symptoms, one must quickly distinguish this disorder from other intracranial pathology such as stroke. A low threshold for screening for hyperglycemia is in order when evaluating patients with acute hemichorea-hemiballismus, even without a known history of diabetes.
